# Arachnoiditis Extending Beyond Operative Site

**DOI:** 10.7759/cureus.32232

**Published:** 2022-12-05

**Authors:** Matthew Tong, Henry Tong

**Affiliations:** 1 Biomedical Engineering, University of Michigan, Ann Arbor, USA; 2 Physical Medicine and Rehabilitation, Providence Hospital, Southfield, USA

**Keywords:** arachnoiditis, discectomy, laminectomy, lumbar disc herniation, lumbar disc herniation surgery

## Abstract

Lumbar epidural fibrosis may occur after a lumbar discectomy, replacing normal epidural fat with non-physiologic scar tissue, binding the dura and nerve roots to the surrounding structures, and causing arachnoiditis. Lumbar arachnoiditis occurs in 6%-16% of postoperative surgeries in the lumbar region, usually at the site of the laminectomy into the spinal canal. This case report covers a 35-year-old male patient who underwent a discectomy with resulting arachnoiditis multiple levels cranial of the site of laminectomy. We illustrate the first reported case of diffuse arachnoiditis causing residual pain after a lumbar discectomy.

## Introduction

Lumbar epidural fibrosis may occur after a lumbar discectomy, replacing the normal epidural fat with non-physiologic scar tissue, binding the dura and nerve roots to the surrounding structures which cause arachnoiditis [1]. Lumbar arachnoiditis occurs in 6%-16% of postoperative surgeries in the lumbar region [2]. Risk factors of arachnoiditis include traumatic taps, blood in cerebrospinal fluid, paresthesias, and injection of neurotoxic or neuro-irritants into the subarachnoid space [3]. Hurme et al. found that five years after lumbar discectomy, an increased amount of scar tissue correlated with poor results [4]. This usually occurs at the site of the laminectomy into the spinal canal [5]. This report covers the case of a patient who had an L5-S1 discectomy with resulting arachnoiditis multiple levels cranial of the operative site.

## Case presentation

A 34-year-old male presented to a private practice Physical Medicine and Rehabilitation Clinic with primarily lower back pain and discomfort. The patient had a history of a fall into a ditch while working about 2.5 years prior, causing lower back pain. His past medical history included irritable bowel syndrome and a right shoulder labral tear for which he had surgery about 20 months prior. He denied any history of Cushing’s disease, asthma, or any disorder that would require prolonged corticosteroid exposure. He had no history of lumbar puncture or meningitis.

**Figure 1:**
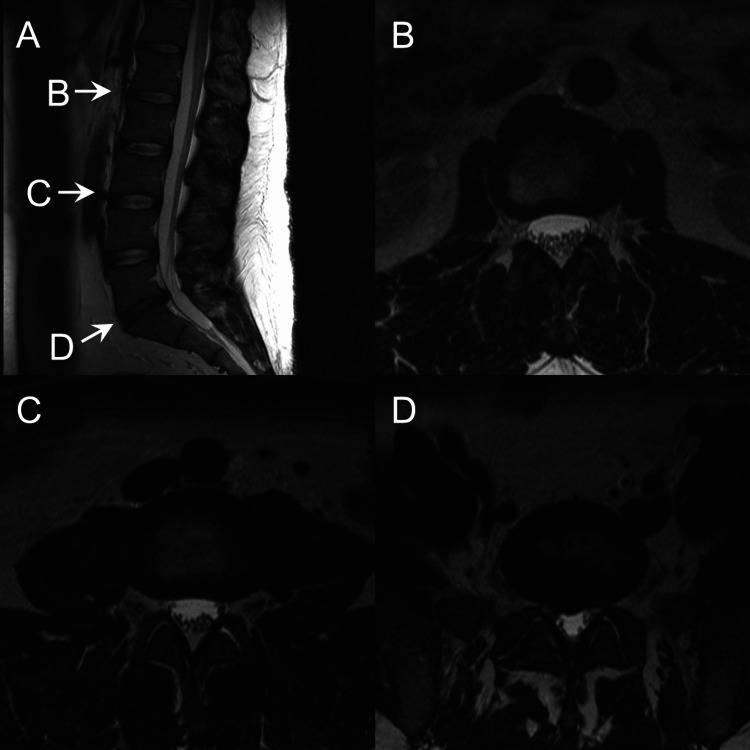
T2 weighted images of the lumbar spine before surgery, (A) midline sagittal view, (B) L1-2 intervertebral disc space axial view, (C) L3-4 intervertebral disc space axial view, and (D) L5-S1 intervertebral disc space axial view.

About 18 months after the fall, a magnetic resonance imaging (MRI) scan (Figures 1A-1D) of the lumbar spine without contrast noted an L5-S1 right herniation possibly contacting the right S1 nerve root. There was no evidence of arachnoiditis. Six months after the MRI scan, he underwent an L5-S1 microdiscectomy and laminectomy with improved, but residual, lower back pain and spasms. A dural leak occurred and was repaired during the microdiscectomy. No infection occurred during the perioperative period. The patient was given general anesthesia, but the exact medication used was not reported.

**Figure 2:**
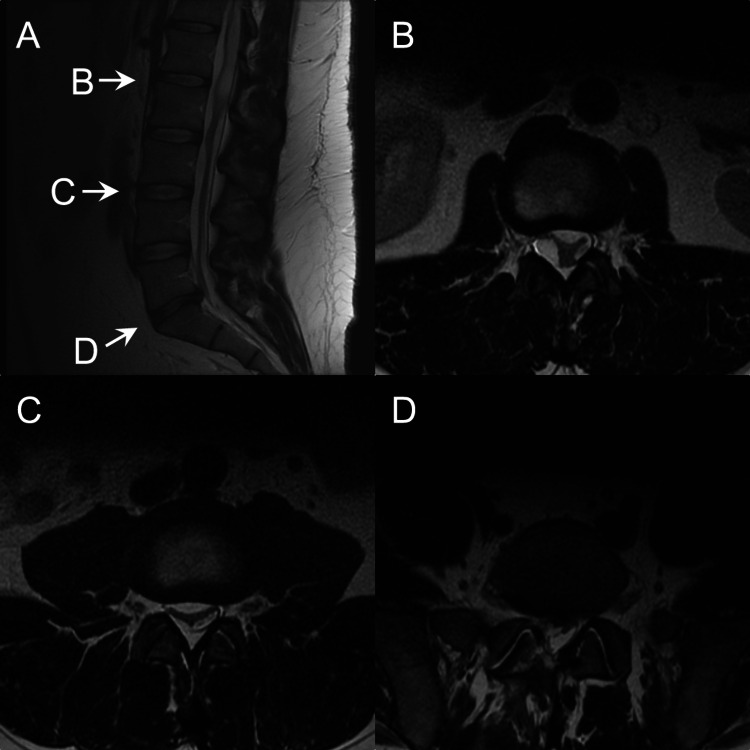
T2 weighted images of the lumbar spine after surgery, (A) midline sagittal view, (B) L1-2 intervertebral disc space axial view, (C) L3-4 intervertebral disc space axial view, and (D) L5-S1 intervertebral disc space axial view.

Three months after the surgery, and nine months after the first MRI scan, a second MRI (Figures 2A-2D) of the lumbar spine with and without contrast showed evidence of nerve clumping extending from the L5-S1 level up to the L1-2 level, suggestive of arachnoiditis. There was no significant disc herniation at any of the lumbar levels. Based on T1 weighted images post-contrast, scar tissue was only present at the laminectomy site at the L5 level in the operative tract, but there were no pathologic signs of contrast enhancement in the entire spinal canal.

## Discussion

Arachnoiditis has been known to occur after lumbar discectomy operations [1]. However, this has only been reported to occur locally at the level of the surgery. This is the first case report to demonstrate that arachnoiditis can spread multiple lumbar levels from the site of the surgery.

The exact cause for this excessive scar tissue is unknown, so there is no widely accepted protocol to help prevent its development. However, the known risk factors of arachnoiditis include traumatic taps, blood in cerebrospinal fluid, paresthesias, and injection of neurotoxic or neuro-irritants into the subarachnoid space [3]. In this case, the dural leak during the microdiscectomy increased the risk of arachnoiditis formation. The presence of arachnoiditis is important as the excess scar tissue can cause a reduction in patient quality of life and lead to poor results [4].

Knowing the spinal levels affected by arachnoiditis or scar tissue is also important for clinical care. First, the spinal levels affected by arachnoiditis can help the clinician determine what neurologic levels could be affected. This will help determine where the patient could have spinal pain as well as symptoms further away from the spine to the limbs, based on knowing the dermatome and myotome nerve distribution. Also knowing the spinal levels affected can help guide treatment. For example, for specific physical therapy approaches, including neural mobilization, the treatment is directed at specific peripheral nerves and their root levels which would be tailored to the levels affected. The femoral nerve targets the L2-3 level, sciatic nerve targets the L4-S1 levels. Similarly, spinal injections with epidurals are also focal and would need to be tailored to the levels affected. 

In this case, knowing that so many root levels are affected, it may be decided to pursue treatments that could help multiple root levels at one time such as medications that calm the nerves (gabapentin, duloxetine, ketamine infusion) [6]. Similarly, a spinal cord stimulator implant could be considered a treatment option that helps multiple lumbar levels at the same time.

This patient received several medications that did not adequately help his pain including hydrocodone-acetaminophen, oxycodone-acetaminophen, tapentadol, morphine, fentanyl patches, methadone, and hydromorphone. Currently, the patient uses meloxicam (7.5 mg daily) and buprenorphine/naloxone (8/2 mg every 12 hours) for baseline pain control. IV ketamine infusions are given as needed for flare-ups. On this regimen, the patient's pain generally ranges from 3 to 6 out of 10.

The differential diagnosis includes epidural lipomatosis. However, this is unlikely since epidural lipomatosis is an extradural fat tissue that pushes in on the thecal sac, causing central canal stenosis [7]. The MRI scan done six months before surgery (Figures 1A-1D) showed no signs of central canal stenosis or epidural lipomatosis, and the MRI scan done three months after surgery showed diffuse nerve clumping from the L1-S1 levels without central stenosis (Figures 2A-2D). Epidural lipomatosis also progresses slowly and is associated with the onset of neurologic symptoms of which this patient has none, even six years post-MRI [8]. Therefore, the finding of this case being arachnoiditis is most likely because arachnoiditis is known to occur after lumbar surgery [1].

## Conclusions

We illustrate the first reported case of diffuse arachnoiditis causing residual pain after a lumbar discectomy. There is no widely accepted protocol to help prevent the development of arachnoiditis beyond standard operating procedures. We believe that the possibility of diffuse arachnoiditis should be considered after a lumbar discectomy as its occurrence has resulted in residual lower back pain and spasms causing disability for the patient.
